# UV-Crosslinked Poly(*N*-isopropylacrylamide) Interpenetrated into Chitosan Structure with Enhancement of Mechanical Properties Implemented as Anti-Fouling Materials

**DOI:** 10.3390/gels10010020

**Published:** 2023-12-25

**Authors:** Isala Dueramae, Fumihiko Tanaka, Naoki Shinyashiki, Shin Yagihara, Rio Kita

**Affiliations:** 1 Micro/Nano Technology Center, Tokai University, Hiratsuka 259-1292, Japan; 2 Metallurgy and Materials Research Institute, Chulalongkorn University, Bangkok 10330, Thailand; 3 Department of Polymer Chemistry, Graduate School of Engineering, Kyoto University, Kyoto 615-8510, Japan; ftanaka@kmj.biglobe.ne.jp; 4 Department of Physics, Tokai University, Hiratsuka 259-1292, Japan; 8gihara.wsal@gmail.com

**Keywords:** chitosan, poly(*N*-isopropylacrylamide), thermo-reversible gelation, interpenetration polymer network, viscoelastic property, anti-fouling materials

## Abstract

High-performance properties of interpenetration polymer network (IPN) hydrogels, based on physically crosslinked chitosan (CS) and chemically crosslinked poly(*N*-isopropylacrylamide) (PNiPAM), were successfully developed. The IPN of CS/PNiPAM is proposed to overcome the limited mechanical properties of the single CS network. In this study, the viscoelastic behaviors of prepared materials in both solution and gel states were extensively examined, considering the UV exposure time and crosslinker concentration as key factors. The effect of these factors on gel formation, hydrogel structures, thermal stabilities of networks, and HeLa cell adhesion were studied sequentially. The sol–gel transition was effectively demonstrated through the scaling law, which agrees well with Winter and Chambon’s theory. By subjecting the CS hydrogel to the process operation in an ethanol solution, its properties can be significantly enhanced with increased crosslinker concentration, including the shear modulus, crosslinking degree, gel strength, and thermal stability in its swollen state. The IPN samples exhibit a smooth and dense surface with irregular pores, allowing for much water absorption. The HeLa cells were adhered to and killed using the CS surface cationic charges and then released through hydrolysis by utilizing the hydrophilic/hydrophobic switchable property or thermo-reversible gelation of the PNiPAM network. The results demonstrated that IPN is a highly attractive candidate for anti-fouling materials.

## 1. Introduction

Hydrogels are hydrophilic polymer networks that can retain a large amount of water within their structure without dissolving or losing their three-dimensional (3D) shape. This is possible due to their chemically or physically crosslinked networks. Both natural and synthetic polymers have gained significant attention in various biomedical applications due to their biocompatibility, making them similar to living tissues [1,2,3]. Additionally, hydrogels have been utilized in fields such as 3D printing [4], wearable devices [5], actuators [5,6], and energy storage devices [7,8]. However, many applications frequently encounter issues with the mechanical properties of hydrogels, both in dry and swollen states. This is particularly true for single-network hydrogels. In a recent study, we presented a simple yet effective approach to enhance the mechanical properties of single-network hydrogels. This was achieved by introducing a second network structure, using the interpenetrating polymer networks (IPNs) method [9]. The IPNs involve the creation of two or more intertwined polymer networks that are not chemically bonded to each other [9,10]. Numerous studies have been conducted on preparing IPN hydrogels by using various hydrophilic polymers or their precursors. The main classes of these polymers include natural polymers and their derivatives (such as polysaccharides and proteins), as well as synthetic polymers that contain hydrophilic functional groups like -COOH, -OH, -CONH_2_, -SO_3_H, amines, and -R_4_N^+^, and ether [10].

Chitosan (CS) is a polysaccharide obtained through the deacetylation process of chitin. Chitin is a linear copolymer consisting of linked *β*(1 → 4) glucosamine (2-amino-2-deoxy-*D*-glucose) and *N*-acetyl-*D*-glucosamine (2-acetamido-2-deoxy-*D*-glucose). The remarkable characteristics of excellent biocompatibility and admirable biodegradability, coupled with ecological safety and low toxicity, along with versatile biological activities like antimicrobial activity and low immunogenicity, have opened up numerous opportunities for further development [11]. CS hydrogels can be chemically constructed using crosslinking reagents such as glutaraldehyde. Alternatively, CS gels can be formed simply through physical crosslinking, which occurs through H-bonds, ionic bonds, dipole interactions, or hydrophobic associations with hydrophobic substituted glucose units, similar to acetylated substituted dextran-based polysaccharides [12,13]. Although these physical gels might dissociate when deformed, they still provide an energy dissipative mechanism that helps prevent sudden shocks or impacts that could harm materials in the application device [14].

Poly(*N*-isopropylacrylamide) (PNiPAM) is a widely recognized thermo-responsive polymer that undergoes a reversible conformational transition from a coil to a globule state at a lower critical solution temperature (LCST) of approximately 32.0 °C in an aqueous solution. This temperature is relatively close to the human body temperature [15]. Similarly, the PNiPAM hydrogel is a well-known temperature-sensitive gel that exhibits a volume phase transition temperature (VPTT) at around 34 °C [16]. Below this temperature, the gel undergoes swelling due to the formation of strong hydrogen bonds between the hydrophilic amide groups and water. As the temperature is raised, the hydrogel undergoes a shrinkage due to the disruption of hydrogen bonds and the significant strengthening of hydrophobic interactions among the hydrophobic groups. This polymer chain collapse results in the hydrogel network’s phase transition [17,18]. Both linear and hydrogel structures possess attractive features that make them suitable biomedical polymers. These features include biocompatibility and non-toxicity and have garnered significant attention in diverse applications [19,20,21,22,23].

CS/PNiPAM hydrogels have garnered significant attention due to their exceptional properties for biomedical and related applications [20,21,24,25,26,27,28,29]. However, it is worth noting that these applications have typically been explored without thoroughly examining the hydrogels’ mechanical properties [20,21,24,25,26,27]. For instance, a temperature-sensitive polymer consisting of palmatine (PA)-loaded cysteine (Cys)-modified chitosan (Cs) grafted with PNIPAM (Cs-Cys-PN/PA) was prepared. This polymer was developed as a fluorescent probe for living cell temperature sensors and for its antibacterial application. The materials have been successfully polymerized, exhibiting aggregation-induced emission enhancement (AIEE) properties, resulting in a reversible hydrogel formation in an aqueous solution. These hydrogels demonstrate low cytotoxicity and do not require any mechanical reinforcement [24]. Excellent mechanical properties are crucial in biomedical fields. However, the inherent mechanical weakness of hydrogels is a significant drawback that limits their applications. Liu et al. evaluated the mechanical properties of the hydrogels via a rheological analysis. The addition of chitosan to alginate-g-poly(*N*-isopropylacrylamide) (Alg-PN_31_-77%) resulted in an enhancement of the elastic modulus, making it approximately 3.5 times larger than that of the Alg-PN_31_-77% copolymer hydrogel. However, the magnitudes were reported unsatisfactorily to be about 180 Pa [20]. To fine-tune the mechanical behavior, crosslinking agents such as glutaraldehyde were commonly used to crosslink chitosan chemically. Additionally, linear PNiPAM was initially functionalized before grafting onto the chitosan structure. As mentioned in previous reports, the hydrogel formation was undoubtedly complex.

We recently demonstrated a facile and versatile approach to constructing the sequential IPN of CS and PNiPAM. This is typically achieved by swelling the chitosan hydrogel in a solution containing a mixture of *N*-isopropylacrylamide monomer (NiPAM), *N*,*N*-methylenebisacrylamide crosslinker (BIS), and 2-hydroxyl-4-(2-hydroxyethoxy)-2-methylpropiophenone photo-initiator (I2965). These components are dissolved in two different solvents: deionized water and ethanol. The CS hydrogel is formed through physical crosslinking. The 3D network of PNiPAM is crosslinked using ultraviolet light (UV), as shown in Supplementary Figure S1. Theoretically, the preparation method, polymer concentration, and concentration of crosslinking agent can significantly contribute to the development of new hydrogel systems and increase the practical value [30]. Therefore, controlling the degree of the crosslinking agent in the PNiPAM structure is expected to enhance the mechanical properties of the single CS network, addressing the gaps in previous research. Further studies were conducted to analyze the rheological and viscoelastic properties, network structure formation, microstructure, thermal properties, and swelling properties. Additionally, HeLa cell adhesion was examined to ensure that the designed material can be used as an anti-fouling material. The HeLa cells that were selected demonstrated higher protein quantification in comparison to other cells [31]. These cells were also utilized as an indicator of protein adsorption. Protein adsorption plays a crucial role in assessing the anti-fouling properties, as it represents the initial phenomenon of cell adhesion to the material’s surface [3]. These efforts aim to provide valuable insights and advancements in this field.

## 2. Results and Discussion

### 2.1. UV-Induced Sol-to-Gel Transition of PNiPAM Solution

Before constructing the IPN network, the conditions for PNiPAM gel formation were optimized. Unlike other systems where temperatures varied from around 50 °C to as high as 75 °C [17,32], the IPN preparation was operated explicitly at room temperature. Therefore, the gelation time needed to be identified to ensure the formation of a three-dimensional network. Several techniques, such as light scattering (LS) [12,33], beam diffraction [13], and rheology [34,35,36], have been widely used to elucidate the mechanism of material gelation and its molecular structure. In this work, the rheological technique was used to investigate the crosslinking behavior due to its direct correlation with the evolving physical and mechanical properties of the system during the crosslinking process.

**Figure 1:**
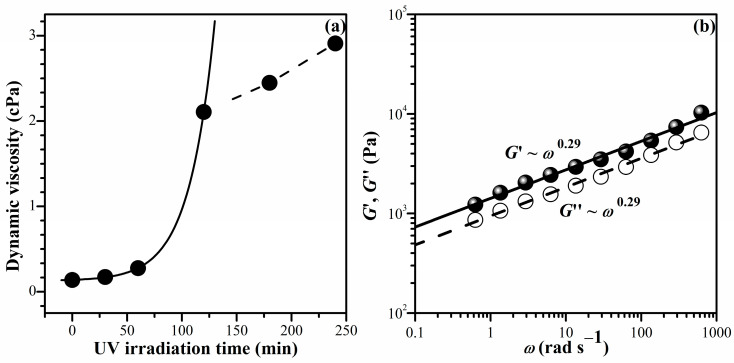
Investigation of sol–gel transition for PNIPAM solutions containing a BIS concentration of 1.45 wt% (0.01 M) measured at room temperature: (**a**) zero-shear viscosity (*η*_0_) vs. UV irradiation time (*t*); (**b**) the frequency-dependent behavior of the moduli, i.e., *G*′~*G*″~*ω^n^* at *t* = 120 min.

Figure 1a illustrates the relationship between the zero-shear viscosity (*η*_0_), obtained from Supplementary Figure S2, and UV irradiation time (*t*) for the mixture of NiPAM (1 M) and BIS (0.01 M) containing I2965 (0.02 M). The *η*_0_ gradually increased with the increasing UV irradiation time within 0–60 min. Subsequently, it exhibited a rapid increase after exposure to UV radiation for more than 60 min. The relationship between $\eta_{0}$ and *t* distinctly changed within a UV irradiation time of 120–240 min, exhibiting linearity. The gel point (*t*_g_) is tentatively identified as the intersection at 120 min. In another experiment, the gelation point was also associated with the parallel lines of shear and loss modulus (*G*′ and *G*″), as shown in Figure 1b. As gelation proceeds beyond the sol–gel transition, log–log plots of *G*′ and *G*″ versus angular frequency produce a parallel line at *t* = 120 min, with the corresponding relaxation exponent, *n*, for *G*′~*G*″~*ω^n^* [37]. The slope, *n*, was found to be 0.29, which corresponds to the chemical gel in previous work (*n* < 0.5) [38]. Therefore, a UV irradiation time of 120 min is considered to be an appropriate condition for initiating the formation of the gel network of PNiPAM in our experimental condition. However, to achieve a fully developed three-dimensional network of PNiPAM, subsequent material construction was carried out using a UV irradiation time of 180 min.

### 2.2. Effect of Crosslinker Concentration on Viscoelastic and Mechanical Behaviors of Single and Interpenetrating Polymer Networks

**Figure 2:**
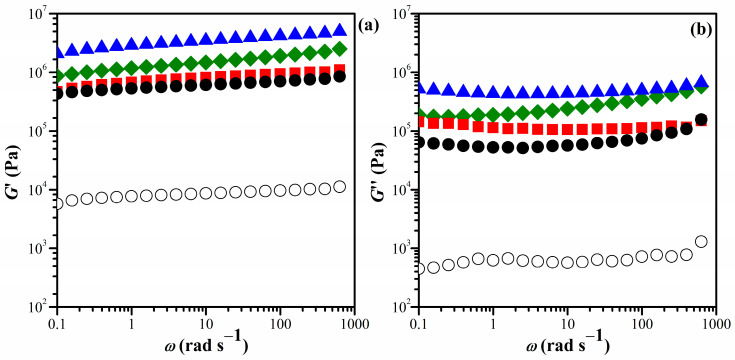
Frequency dependence of the (**a**) *G*′ and (**b**) *G*″ of the hydrogels at 25 °C, with a BIS crosslinker content of 0 wt% (CS hydrogel) (○), 1.45 wt% IPN_E_1 (●), 2.65 wt% IPN_E_2 (■), 5.16 wt% IPN_E_3 (▲), and 21.42 wt% IPN_E_4 (♦).

The composition of the starting materials and the abbreviations for the resulting hydrogels are listed in Table 1. The viscoelastic properties of the hydrogels were analyzed by studying the frequency-dependent behavior of the shear modulus, *G*′; loss modulus, *G*″; and tan *δ* at room temperature. As shown in Figure 2 (and Supplementary Figures S3–S6, in detail), the *G*′ was significantly higher than the *G*″, and both *G*′ and *G*″ exhibited slight variations with increasing frequency for all IPN hydrogels. This indicated that the hydrogels have a stable network with elastic characteristics owing to the high molecular intra- and/or inter-actions level. The results demonstrate that the viscoelastic properties of chitosan are significantly enhanced by incorporating the PNiPAM chain structure.

**Figure 3:**
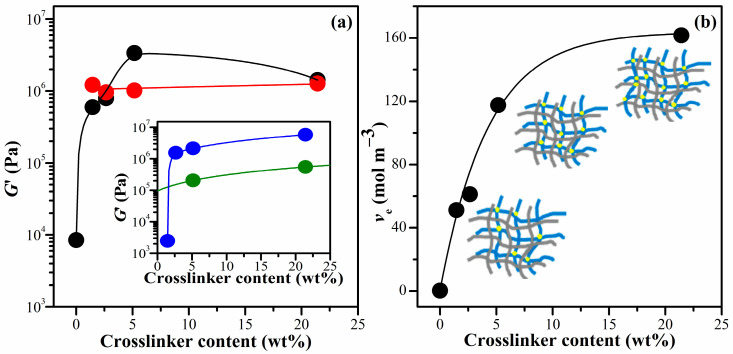
(**a**) Shear modulus, *G*′, obtained at 1 Hz (6.28 rad s^−1^) with various crosslinker contents. *G*′ of IPNs prepared in ethanol solution (IPN_E_ system) (●) and IPN prepared in aqueous solution (IPN_W_ system) (●). The inset shows *G*′ of PNiPAM hydrogels prepared in ethanol solution (PNiPAM_E_) (●) and in aqueous solution (PNiPAM_W_) (●) as a function of crosslinker content (Table 1 involves 5.16 wt% BIS only for PNiPAM_E_ and PNiPAM_W_ systems). (**b**) The crosslinking density, *v*_e_, as a function of crosslinker content for IPN_E_ system. The inset shows a schematic IPN network model in which gray and blue networks represent the CS and PNiPAM chains, respectively. Yellow circles are the crosslinking points. Curves are drawn to guide eyes.

At a single frequency of 1 Hz (6.28 rad s^−1^), the *G*′ of all the hydrogels was compared as a function of the crosslinker content, as shown in Figure 3a. The *G*′ of CS hydrogel increased significantly with an increase in the crosslinking agent. This can be attributed to the inherent structure of pure PNiPAM, as illustrated in the inset of Figure 3b, where the *G*′ of pure PNiPAM prepared in an ethanol solution also showed a significant increase with an increase in the crosslinking agent. These hydrogels prepared in ethanol (IPN_E_ systems) provided a higher value of *G*′ compared to those prepared in deionized water (IPN_w_ systems). Interestingly, the *G*′ of the IPN_E_ system showed a substantial concentration dependency for the crosslinker content, whereas that of the IPN_w_ system remained almost constant value even when the crosslinker increased. This might be due to the excess crosslinker content above 0.01 M (1.45 wt%) in the aqueous solution, where the conversion of NiPAM to PNiPAM remained almost constant regardless of the crosslinking agent used in this polymerization condition. The *G*′ of the IPN hydrogels (0.6–3.37 MPa) was approximately 70-to-400 times higher than that of the CS hydrogel. Previous studies have shown that the *G*′ value for the hydrogel containing polyvinyl pyrrolidone/carboxymethyl cellulose at a 1:4 ratio was approximately 0.43 MPa [39]. On the other hand, the *G*′ of Salecan and poly(2-acrylamido-2-methylpropanesulfonic acid-co-N-hydroxymethyl acrylamide) semi-IPN hydrogels ranged from 0.27 to1.14 kPa [40]. This indicates a relatively high modulus for the hydrogels prepared in this study.

The theory of rubber elasticity states that the elastic modulus of a soft polymer network is directly proportional to the density of the crosslink points. In the case of a phantom network, the *G*′ of gels is determined by the crosslinking density (*v*_e_) and the extent of solvent swelling, as depicted in Equations (1) and (2) [40]:

(1) $$G^{\prime }=(1-\frac{2}{\phi })v_{e}RTv_{2}^{2/3}$$

(2) $$v_{2}={\left[1+\frac{\left(q_{F}-1\right)\rho }{d}\right]}^{-1}$$

where *v*_2_ is the volume fraction of crosslinked polymer in the hydrogel; *R* and *T* are the gas constant and absolute temperature, respectively; *ϕ* is the functionality of the crosslinks. The *q*_F_ represents the mass of swollen gel in equilibrium divided by the constant weight of the hydrogel after solvent evaporation. This value increases as the crosslinker content increases and reaches its maximum at 2.65 wt% crosslinker. The details of this phenomenon are discussed in Section 2.5, specifically referred to as the equilibrium swelling ratio (*SR*_e_). The symbol *ρ* represents the polymer density, and *d* represents the density of the solvent [40]. The crosslink densities, *v*_e_, were calculated from Equations (1) and (2), and they are depicted in Figure 3b for the IPN_E_ system. At the concentration of 21.4 wt% of BIS content (IPN_E_4), the *v*_e_ increased further, but the *G*′ decreased, suggesting a high degree of brittleness in the IPN_E_4 structures. It has been reported that adding an excess amount of crosslinking agent enhances the density of the CS network and decreases the flexibility of the chains due to the reinforced chain entanglements [41].

To gain insights into the influence of BIS concentration on the viscoelastic properties of liquid and solid gels, we scrutinized important parameters such as the relaxation exponent and gel strength of the materials. Following the Winter–Chambon criterion [42], the power law relation is also evident in dynamic mechanical experiments, as expressed below:

(3) $$G^{\prime }=\frac{G^{\prime \prime }}{\tan\delta }=S\omega^{n}\Gamma \left(1\;-n\right)\cos\delta$$

where *Γ*(1 − *n*) represents the gamma function; *n* denotes the relaxation exponent; and *δ* is the phase angle that remains independent of frequency but is proportional to the relaxation exponent, given by *δ = n*π/2. Additionally, *S* represents the gel strength parameter, which relies on the crosslinking density and molecular chain flexibility.

The power law of mechanical behavior represents the self-similar (fractal) structure of clusters at the gel point (*GP*) [37,43]. The statistical self-similarity of a polymer is quantitatively represented by a fractal dimension, *d_f_*, which describes the relation between the mass of a molecular cluster (*M*) in the network to its radius (*R*) through the expression of *R^d^_f_*~*M*. Muthukumar established an expression for the relaxation exponent, *n*, in terms of the *d_f_* for polydisperse material, where the excluded volume effect of the polymer chain is completely screened. The equation is given as follows [43]:

(4) $$n=\frac{d\left(d+2-2d_{f}\right)}{2\left(d+2-d_{f}\right)}$$

where *d* (=3) is the spatial dimension. As mentioned earlier, the values of the relaxation exponent, *n*, are determined by analyzing a log–log plot and the scaling relation of the *G*′ and *G*″. The *d_f_* values are obtained in the range of 2.22–2.44 for IPN_E_, which is close to 2.50 of complete screening of excluded volume interactions. It indicates the development of a tight and compact network structure [44].

**Figure 4:**
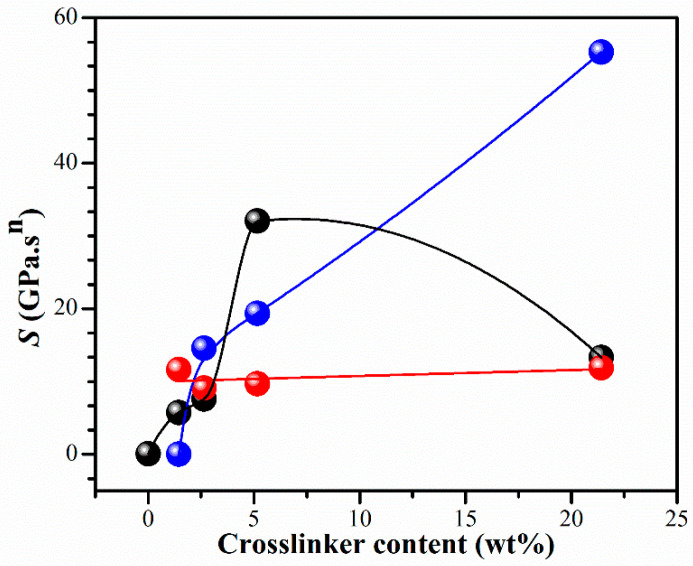
Gel strength, *S*, as a function of the BIS concentration of hydrogels for IPN_E_ system (●), IPN_w_ system (●), and PNiPAM_E_ system (●). Curves are drawn to guide eyes.

The effect of different amounts of BIS on the gel strength, *S*, in Equation (3) is shown in Figure 4. When ethanol was used as a solvent, a clear dependence of the *S* value on the crosslinker concentration was observed. The gel strength, *S*, of CS hydrogel was outstandingly enhanced with the crosslinker content. Interestingly, the IPN_E_3 showed a synergistic character resulting from the crosslinking density. At low concentrations of BIS, the gel network becomes fragile and susceptible to additional crosslinks, resulting in an increase in the strength parameter, *S*. However, at the highest concentration of BIS, the strength of the network is primarily influenced by polymer entanglements, and the impact of additional crosslinks on the gel strength parameter is not as significant, as it is at lower levels of polymer crosslinking. These investigations have shown that the parameter *S* is sensitive to changes in the strand length between crosslinks. When the strand length shrinks (e.g., due to increased BIS concentration), the crosslinking density increases, resulting in a “harder” gel with a higher *S* value.

### 2.3. Temperature Dependence on Mechanical Stability of Swollen Hydrogel Networks

**Figure 5:**
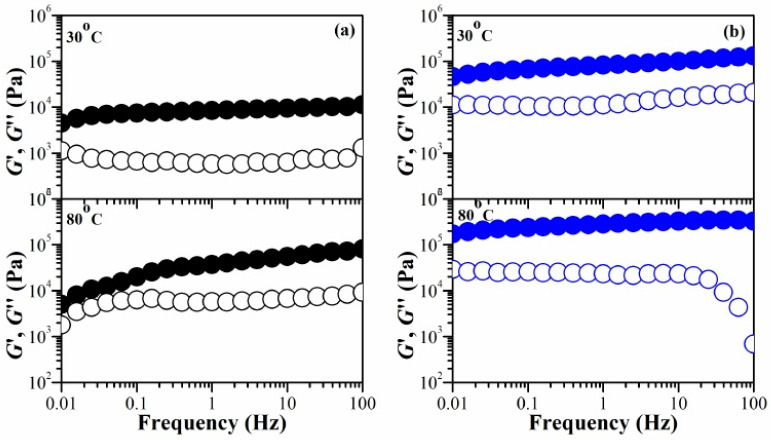
The frequency dependence on the *G*′ and *G*″ of (**a**) CS (chitosan) and (**b**) IPN_E_3 hydrogels: *G*′ (filled circles) and *G*″ (hollow circles) at different temperatures.

Hydrogels typically contain a significant amount of moisture, which results in poor mechanical properties. This is due to the high degree of swelling and low density of the polymer chains. Consequently, this poses a significant challenge for their practical application. We manipulated the water content (%) within the hydrogels to assess their mechanical stability in their swollen state. Figure 5 (and Supplementary Figure S7, in detail) illustrates the temperature influence on the *G*′ and *G*″ for the chitosan hydrogel, comparing it with IPN_E_3 hydrogel in the 30–80 °C temperature range. In the case of the chitosan hydrogel (Figure 5a), the slope of log *G*′ vs. log *ω* increased with the temperature rise. For example, the *G*′ decreases more rapidly as the frequency decreases at 80 °C, compared to the other temperatures. The difference between the *G*′ and *G*″ at low frequency became smaller as the temperature increased from 30 to 80 °C. At 80 °C, a crossover between the *G*′ and *G*″ occurred at approximately 0.06 Hz. On the other hand, the crossover of the *G*′ and *G*″ was not observed for IPN_E_3 hydrogel (Figure 5b). The network structure of chitosan relies on several key interactions: hydrophobic forces, molecular entanglements, and secondary forces such as ionic and hydrogen bonding. These interactions become more time-sensitive under stress. When we decrease the testing frequency, which corresponds to longer experimental durations, the elastic property of the gel, represented as *G*′, decreases, and its viscous nature, *G*″, increases. At high temperatures and low frequencies, the *G*′ and *G*″ become more similar, eventually crossing over. At this point, the *G*″ takes over, indicating a shift from a solid-like to a liquid-like behavior due to a thermal disruption in the physical network.

The IPN_E_3 exhibits a stable network when subjected to stress at varying temperatures. This characteristic of our system can be suggested for utilization in the development of shape-memory hydrogels. Applying temperature or stress can cause the permanent shape of IPN_E_3 to disengage, transforming it into a temporary shape. This temporary shape can be restored by cooling the deformed sample while under load or reducing stress. Shape recovery occurs when the gel in its temporary shape is reheated and/or when a sufficiently large stress is applied to break the physical network. From a molecular perspective, the shape memory cycle regulates the relaxation times of the temporary shape through the amplitude of stress and/or temperature.

### 2.4. Chemical Structure of Single and IPN Hydrogels

**Figure 6:**
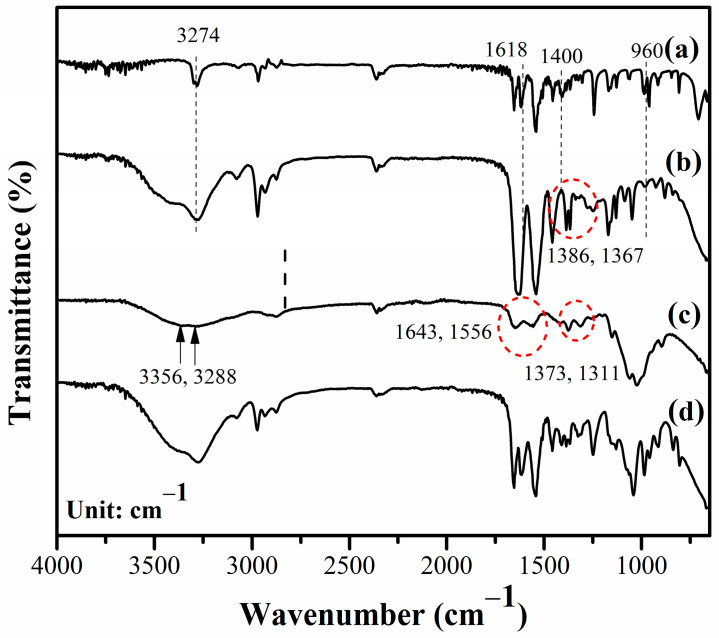
Representative FTIR spectra of the (**a**) NiPAM monomer, (**b**) PNiPAM_E_ hydrogel, (**c**) CS hydrogel, and (**d**) IPN_E_3 hydrogel.

We assessed the chemical structures of the materials by using Fourier-transform infrared spectroscopy (FTIR). Figure 6 shows the FTIR spectra of the NiPAM monomer, PNiPAM_E_ hydrogel, CS hydrogels, and IPN_E_3. Several changes became apparent in the FTIR spectrum of the PNiPAM_E_ hydrogel compared to the NiPAM monomer, as illustrated in Figure 6a,b. The sharp peak at 3274 cm^−1^ in the NiPAM monomer, attributed to the N-H stretching vibrations, became significantly broader in the PNiPAM_E_ hydrogel. This broadening was due to overlap with O-H vibrations from alcohol residues in the solvent. The distinctive spectral peaks of the NiPAM monomer at 1618 and 960 cm^−1^, associated with C=C bending and the vinyl group, vanished in the PNiPAM_E_ hydrogel, indicating polymerization proceeds. Moreover, specific peaks related to the isopropyl group vibrations appeared at 1386 and 1367 cm^−1^, shifting to a lower wavelength than the monomer (1400 cm^−1^), suggesting that these isopropyl groups act as crosslinking points. This formation of crosslinking points was supported by interactions between the *tert*-C atom of the side isopropyl group and the main chain isopropyl group, as explained in a previous study [45].

For the CS hydrogel presented in Figure 6c, we found overlapping peaks at 3356 and 3288 cm^−1^, indicating -OH stretching, symmetric N-H vibration, and intermolecular H-bonds between polysaccharide chains. The carbonyl stretching vibration (amide-I), N-H stretching vibration (amide-II), and C-N stretching vibration (amide-III) of CS were observed at 1643, 1556, and 1311 cm^−1^, respectively. Additionally, the symmetrical deformation of the methyl (CH_3_) groups of CS was identified at 1373 cm^−1^, consistent with prior research [46].

The characteristic peaks of the IPN hydrogel indicate the presence of functional groups from both neat hydrogels. Some shifts in peak positions were observed, as shown in Figure 6d. These shifts suggest that the initial reactants could penetrate the CS hydrogel, and upon exposure to UV light, the PNiPAM structure was formed. Further shifts in the wavenumber and band broadening indicate the formation of intermolecular associations between these pure hydrogels, which display the compatible characteristics of both the CS and PNiPAM hydrogels.

### 2.5. Equilibrium Swelling Ratio (SR_e_) of Hydrogel Networks

**Figure 7:**
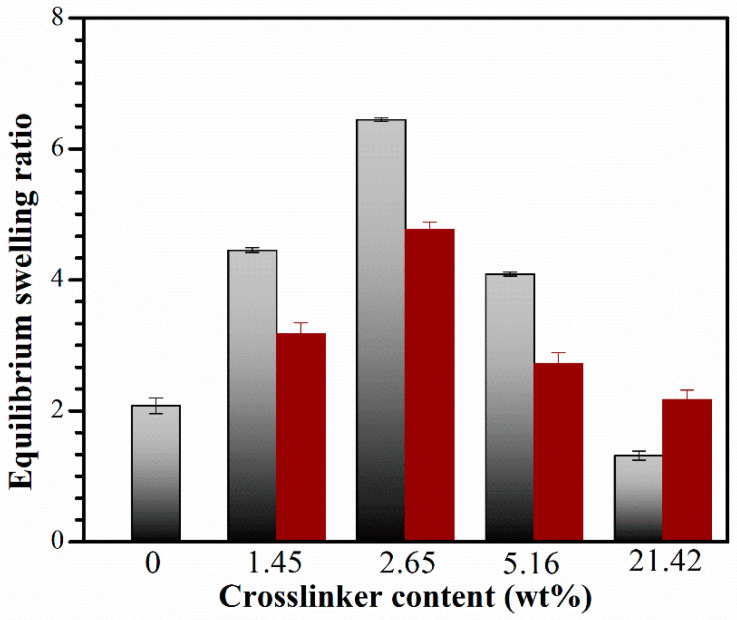
Equilibrium swelling ratio (*SR*_e_) of IPN_E_ system (gray) and IPN_w_ system (wine) with various crosslinker contents. Data are shown as the mean, derived from 3 repeats.

Figure 7 shows the swelling ratio, *SR*_e_, of the CS and IPN hydrogels with varying crosslinker contents, calculated using Equation (5) [47].

(5) $${SR}_{e}\left(g/g\right)=\frac{w_{e}-w_{d}}{w_{d}}$$

where *w*_d_ and *w*_e_ represent the weight of dried hydrogels before being immersed in deionized water and the swollen sample reaching its equilibrium state at room temperature, respectively.

The interpenetration of the PNiPAM network resulted in an increased *SR*_e_ for the CS hydrogel. The CS hydrogel is formed through physical crosslinking, primarily held together by the hydrophobic interactions of the *N*-acetyl group and inter- and/or intra-molecular hydrogen bondings. This results in a polymer network that can easily deform, leading to the releasing or/and de-swelling of the absorbed water. Moreover, the CS network lacks stable crosslinking points, making it capable of absorbing less water than the IPNs, which have a stable network with chemically crosslinked structures.

The hydrophilicity of CS improved when the PNiPAM network was introduced, leading to an increased *SR*_e_ with a rise in the crosslinker content. The network structure exhibited high hydrophilicity, interacting with water molecules most effectively at a crosslinker content of 2.65 wt%, resulting in the highest water uptake. It is well known that the *SR*_e_ of the hydrogels depends not only on the hydrophilic ability of the functional groups but also on the network space of the hydrogels. In general, hydrogels with a higher network space exhibit a higher water content. Therefore, it can be suggested that IPN with a crosslinker content of 2.65 wt% exhibits a greater amount of space between the polymeric chains, allowing for increased absorption of water molecules compared to the IPN with a crosslinker content of 1.45 wt%. However, the *SR*_e_ decreased at crosslinking concentrations exceeding 2.65 wt% due to the increased density of crosslinks and the entanglement of polymer chains within the gel network. This led to a dense structure that hindered further swelling, consistent with our crosslink density findings. As a result, water molecules diffused more slowly into the network, limiting the relaxation of network chains in the hydrogels [48].

The IPN_E_ system exhibited a higher magnitude of *SR*_e_ than the IPN_w_ system. This is because less water could penetrate the dense IPN_w_ structure, whereas the IPN_E_ networks had a more porous structure, illustrating the morphology in FE-SEM results. The mechanism of water absorption will be thoroughly investigated in our future research. The irregular swelling performance is distinct from that of conventional gels. Previous investigations into the mechanism have shown that the equilibrated swelling ratio in good solvents exhibits a negative correlation with the crosslink density [49]. Interestingly, this phenomenon is consistent with findings from previously reported systems [50,51,52,53], such as a series of polyacrylate hydrogels, including poly(methyl acrylate), poly(benzyl acrylate), and poly(methoxyethyl acrylate) [51]. It has been suggested that the mechanism behind the swelling of this IPN system is not yet clearly understood at this moment. However, extensive research has been conducted on the mechanisms and kinetic phenomena associated with swelling behaviors. Moreover, additional experimental evidence will be further validated using dielectric relaxation spectroscopy in the near future.

### 2.6. Microstructure of the CS and IPN Hydrogels

**Figure 8:**
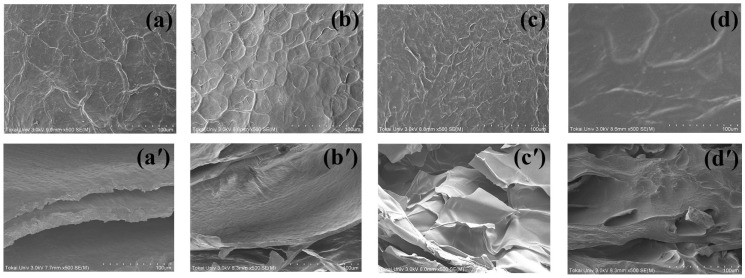
Representative SEM images showing the (**a**–**d**) surface and (**a′**–**d′**) cross-sectional morphology of IPN hydrogels with different crosslinking agents: (**a**,**a′**) 0 wt% CS hydrogel, (**b**,**b′**) 1.45 wt% IPN_E_1, (**c**,**c′**) 5.16 wt% IPN_E_2, and (**d**,**d′**) 21.42 wt% IPN_E_4.

We used FE-SEM to examine the surface and cross-sectional structures of the fresh hydrogels. Figure 8 illustrates the morphologies of CS and IPN hydrogels with different BIS concentrations. The CS hydrogel exhibited a network of interconnected fibers with both coarse and smooth surfaces, consistent with previous findings [54]. Such surface structures are typical of polyelectrolyte components like CS and sodium alginate, as well as their mixtures [55]. When the PNiPAM network was added, the surface of the CS hydrogel became smoother and more uniform as the BIS crosslinker content increased. The roughness of the hydrogel’s surface played a crucial role in altering its morphology. A smoother surface enhances the anti-fouling properties of hydrogel materials, making them more resistant to unwanted adhesion (and Supplementary Figure S8, in detail).

Cross-sectional images of the samples revealed different structures based on the BIS concentration. The CS hydrogel exhibited a well-defined lamellar structure due to the system’s homogeneity, allowing the NiPAM solution to penetrate easily. In the IPNs, a smooth and dense structure with irregular pores was observed, indicating excellent compatibility between the two polymers. The lamellar layer of the CS hydrogel was replaced by the PNiPAM network, creating a porous structure ideal for absorbing wound fluid and facilitating oxygen supply, which promotes faster wound healing [56]. Furthermore, it is a useful material for loading and releasing preservative substances in food-processing applications [57]. The IPN structure becomes denser with a higher crosslinker content, resulting from the increased crosslink density of PNiPAM within the CS network. Moreover, this material finds application in food processing for loading and releasing preservatives [57]. With a higher crosslinker content, the IPN structure became denser due to the increased crosslink density of PNiPAM within the CS network. This homogeneity confirmed the compatibility between CS and PNiPAM, contributing to the high mechanical properties observed in the *G*′ results

### 2.7. HeLa Cell Adhesion and Proliferation on the Hydrogels’ Surface

**Figure 9:**
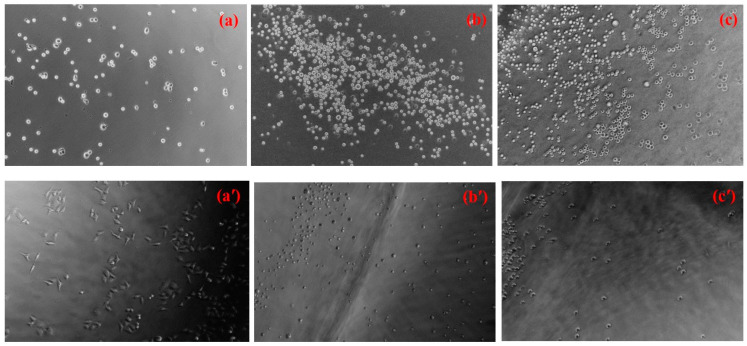
Representative fluorescence images of cells cultured for (**a**–**c**, **top**) 1 h and (**a′**–**c′**, **bottom**) 24 h on the controlled surface of (**a**,**a′**) PS, (**b**,**b′**) CS, and (**c**,**c′**) IPN_E_3.

The biocompatibility of biomaterials can be assessed by examining how cells adhere to them. In this study, HeLa cells, a commonly used immortalized human cancer cell line in research labs worldwide, were used [58]. The morphology of HeLa cells on both the CS and IPN_E_ hydrogels was observed and compared to those on the PS control surface after 1 h of cell seeding, as shown in Figure 9a–c. No significant differences were observed in cell morphology between the control and hydrogel surfaces. However, it was evident that the hydrogel surfaces had more cell colonies than the control surface. The positively charged sites in the CS structure likely enhanced electrostatic interactions with the negatively charged cell membranes and proteins, facilitating cell adhesion to the CS surface [59]. Additionally, the hydrophobic nature of PNiPAM-based IPN might contribute to its cell adhesion properties, as it can switch from a hydrophilic to a hydrophobic state above its VPTT (37 °C), as shown in Supplementary Figure S9. The appearance of the swollen IPN_E_3 in the PBS solution changed from transparent at room temperature to opaque at the incubation temperature (37 °C).

After 24 h of cell culture initiation, HeLa cell proliferation was observed. Cells on the polystyrene (PS) control surface displayed a flattened morphology, indicating strong cell adhesion to the surface, as shown in Figure 9a′. In contrast, cells on the hydrogel surfaces exhibited a rounder morphology, with lower adhesion and less proliferation, as shown in Figure 9b′,c′. Cell populations were counted after detaching from the material surface, revealing that more cells had grown and spread on the PS control’s surface (3.6 ± 0.10 × 10^6^ cells/cm^3^) compared to the hydrogels (Table 1). The hydrogels likely had a significant impact on the strength of interactions with HeLa cells, leading to improved resistance against fouling [60]. The number of adhered cells on the IPN surfaces was slightly lower than on the CS surface, possibly due to the smoother surface, as indicated by the FE-SEM results. In general, cells adhere well to stiff surfaces [61,62], and materials with higher roughness are more prone to fouling, as contaminants tend to accumulate in the “valleys” of rough surfaces [63,64]. These results indicate that IPN_E_3 exhibits satisfactory biocompatibility due to its anti-biofouling properties. Therefore, the IPN_E_3 hydrogel appears to be effective in providing biocompatibility and anti-biofouling characteristics to hydrogel materials in general. Furthermore, the surface of IPN_E_3 underwent a thermal reversal, transitioning from a hydrophobic state at the incubation temperature (37 °C) to its original hydrophilic state at room temperature (hydrophilic/hydrophobic switchable property). This demonstrated the detachment or release of HeLa cells from the surface. In other words, the excellent thermo-reversible gelation of this material offers numerous benefits for various applications. The gel’s strength is significantly enhanced when the IPN_E_3 is swollen and used above the VPTT, as described in Section 2.3 and in the Supplementary Materials. Conversely, it can be reversed to its original transparent state when cooled down below the VPTT.

## 3. Conclusions

We explored a straightforward and versatile method for crafting hydrogel-based interpenetrating polymer networks (IPNs) with several notable advantages, such as enhanced mechanical properties, multifunctionality, and anti-fouling attributes. The process involves a sequential approach in which a CS hydrogel is swollen in a solution containing NiPAM monomer in ethanol or an aqueous solution of a BIS crosslinking agent, employing the I2965 photo-initiator. The PNiPAM network is then established as a crosslinked structure through UV polymerization at room temperature. The gel formation is evident through the sol–gel transition, as confirmed by the scaling law of the Winter and Chambon hypothesis.

Furthermore, we investigated the impact of BIS concentration on the resulting hydrogels’ properties. Increasing the BIS concentration from 1.45 to 21.72 wt% led to improvements in all analyzed rheological and viscoelastic properties of the CS hydrogel. However, exceeding the maximum BIS concentration had a detrimental effect. Higher degrees of crosslinking resulted in the formation of stiffer modules. Among all BIS concentrations, intermediate values of the crosslinking agent (5.16 wt%) demonstrated the best performance.

The incorporation of the PNiPAM network into the CS hydrogel induced the formation of a porous structure, increasing water uptake within the hydrogel networks. Additionally, these materials exhibited significantly greater thermal and mechanical stabilities when exposed to varying temperatures and applied forces, compared to the CS hydrogel, which was vulnerable to thermal disruption. These materials are anticipated to demonstrate exceptional performance characteristics. They can effectively adhere to and eliminate living organisms through the cationic properties of the CS surface while also releasing undesirable molecules through hydrolysis, thanks to the hydrophilic/hydrophobic switchable property of the PNiPAM network.

## 4. Materials and Methods

### 4.1. Materials

All reagents used in this study were analytical grade. Medium molecular-weight CS of 75–85% deacetylation, *N*-isopropylacrylamide, *N*,*N*-methylenebisacrylamide (BIS), and 2-hydroxyl-4-(2-hydroxyethoxy)-2-methylpropiophenone (I2965) were purchased from Sigma-Aldrich. BIS was utilized as a crosslinker, while I2965 served as a photo-initiator. All solvents, including acetic acid, 1,3-propanediol, and ethanol, were purchased from Wako Pure Chemicals, Osaka, Japan.

### 4.2. Characterization of the Samples

#### 4.2.1. Fourier-Transform Infrared Spectroscopy (FTIR)

The chemical structure of the single network and IPN hydrogels was characterized using FTIR analysis, which was conducted on a Horiba FTIR 720 spectrometer equipped with an attenuated total reflectance accessory. The obtained spectra were averaged from 64 scans at a resolution of 4 cm^−1^, within the spectral range of 650–4000 cm^−1^.

#### 4.2.2. Rheological Test

The viscoelastic behavior of fresh and equilibrated samples was evaluated using a TA instrument TRIOS, employing a parallel plate of 25 mm in diameter. The measurements were conducted within the linear viscoelasticity region. The shear modulus (*G*′), loss modulus (*G*″), and loss tangent (tan *δ*) were measured as a function of the frequency, within a frequency range of 0.1–100 rad s^−1^ with 0.01% strain at 25 °C. Each hydrogel was automatically loaded at the normal force of 1.0 N. Thermal and mechanical stabilities of the hydrogels in fresh and swollen states were investigated via the measurement of *G*′ as a function of frequency over the temperature range of 30–80 °C.

#### 4.2.3. Field Emission-SEM (FE-SEM)

The surface and cross-section morphology of the CS and IPN_E_ samples were examined using FE-SEM (Hitachi SU-4800), operated with an accelerating voltage of 3.0 kV and emission current of 10 mA. Before measurement, the fracture surfaces of the materials were sputter-coated with gold.

#### 4.2.4. Measurement of Equilibrium Swelling Ratio (*SR*_e_)

The swelling characteristics of the hydrogels were measured gravimetrically. The dried hydrogels were weighed before being immersed in deionized water at room temperature (*w*_d_). The swollen hydrogels were then removed from the water at regular intervals and weighed after excess water on the hydrogel surfaces was removed using filter paper (*w*_t_). The average value of three measurements was taken for each hydrogel to minimize errors. The water absorption of the hydrogel was continuously measured to allow it to reach its equilibrium swelling value (*w*_e_). The equilibrium swelling ratio (*SR*_e_) was calculated using Equation (5) [46].

(6) $${SR}_{e}(g/g)=\frac{w_{e}-w_{d}}{w_{d}}$$

#### 4.2.5. Cell Adhesion Test

HeLa cells (JCRB, #JCRB9004), an immortal cell line derived from human cervical cancer cells, were cultured in a complete medium (CM). The CM consisted of Dulbecco’s Modified Eagle’s medium supplemented with 2 mM L-glutamine and 10% (*v*/*v*) fetal bovine serum (Biowest, France). After rinsing with phosphate-buffered saline (PBS) at a pH of 7.2, the sub-cultured cells were harvested from a tissue culture polystyrene dish, using a 0.25% (*w*/*v*) trypsin/1 mM-EDTA solution. The cells were then recovered via centrifugation at 500× *g* for 5 min. Subsequently, the cells were seeded on polymer sheets at a density of 1.0 × 10^4^ cells/cm^2^. The polymer sheets, free from any solvent contaminants and unreactive components, were pre-equilibrated in PBS for 2 h before being cultured in complete medium (CM) for 24 h at 37 °C under 5% (*v*/*v*) CO_2_. After being washed with PBS, the medium was replaced with fresh CM, and the cells were imaged using a Nikon BW-S507 fluorescence microscope at Tokai University Imaging Center for Advanced Research. The cell numbers were counted after adding trypsin blue solution to a final 0.2% (*v*/*v*).

## Figures and Tables

**Table 1:** Characteristics of the single gels (chitosan and PNiPAM) and IPN hydrogels.

Name	NiPAM (M)	BIS (M, wt%)	Photo-Initiator /I2959 (M)	Prepared Solvent	Cell Content (10^3^ Cell/cm^2^) ^a^
**Single polymer network**					
**CS (Chitosan)**	0	0	0		4.50 ± 0.54
**PNiPAM_E_**	1	0.04 (5.16)	0.02	Ethanol	N/A
**PNiPAM_W_**	1	0.04 (5.16)	0.02	Water	N/A
**Interpenetrated polymer network of CS/PNiPAM prepared in ethanol**					
**IPN_E_1**	1	0.01 (1.45)	0.02	Ethanol	3.70 ± 0.29
**IPN_E_2**	1	0.02 (2.65)	0.02		3.30 ± 0.50
**IPN_E_3**	1	0.04 (5.16)	0.02		2.40 ± 0.52
**IPN_E_4**	1	0.20 (21.4)	0.02		2.40 ± 0.43
**Interpenetrated polymer network of CS/PNiPAM prepared in water**					
**IPN_w_1**	1	0.01 (1.45)	0.02	Water (DI)	N/A
**IPN_w_2**	1	0.02 (2.65)	0.02		
**IPN_w_3**	1	0.04 (5.16)	0.02		
**IPN_w_4**	1	0.20 (21.4)	0.02		

The subscribed _E_ and _W_ represent the ethanol and deionized water, respectively, as solvents. ^a^ Data are shown as the mean, derived from 3 repeats.

## Data Availability

The data presented in this study are openly available in article.

## Supplementary Materials

The following supporting information can be downloaded at https://www.mdpi.com/article/10.3390/gels10010020/s1, Figure S1. The fabrication process of (a) chitosan hydrogels, (b) PNiPAM hydrogels, and (c) the physical appearance of gels. Figure S2. Rheological measurement of ethanol system of NiPAM monomer and photo-initiator containing BIS concentration of 1.45 wt% at different UV irradiation times. (a) Shear-rate dependence of viscosity; UV irradiation times are 0 min (black), 30 min (red), 60 min (blue), 120 min (yellow), 180 min (olive), and 240 min (cyan). (b) Frequency dependence of G′ (filled circles) and G″ (hollow circles); UV irradiation times are 0 min (black) and 240 min (cyan). Figure S3. Frequency dependence of G′ (filled circles) and G″ (hollow circles) at room temperature for CS hydrogel (black) and PNiPAME hydrogels at various BIS concentrations as 1.48 wt% (red), 2.65 wt% (blue), 5.16 wt% (olive), and 21.42 wt% (yellow). Figure S4. Frequency dependence of the tan *δ* for hydrogels of CS and IPN_E_ series with various crosslinker content of CS hydrogel (0 wt% (○)), IPN_E_1 (1.45 wt% (●)), IPN_E_2 (2.65 wt% (■)), and IPN_E_3 (5.16 wt% (▲)) measured at room temperature. Figure S5. The physical appearance of PNiPAM hydrogels prepared in different solvents after UV irradiation for 3 h: (a) ethanol and (b) deionized water. Insets show the transparency of NiPAM, BIS, and I2965 solution mixtures in both solvents before UV irradiation. Figure S6. The physical appearance of PNiPAME hydrogels prepared in ethanol solvents with a crosslinker content of (a) 1.45 wt%, (b) 2.65 wt%, (c) 5.16 wt%, and (d) 21.42 wt%. Figure S7. The frequency dependence on the G′ (filled circles) and G″ (hollow circles) of (a) CS (chitosan) and (b) IPNE3 hydrogels measured at different temperatures. Figure S8. Frequency dependence on the shear modulus of the swollen state of CS (black) and IPN_E_3 (blue) hydrogels at the temperatures 30 °C (●), 40 °C (■), and 60 °C (♦). Figure S9. The physical appearance for hydrogels of CS (left side) and thermo-reversible behavior of IPNE3 (right side). (a) At room temperature before cell seeding. (b) During the incubation process at 37 °C. (c) Room temperature after incubation process. References [38,65,66,67,68,69,70,71,72,73] are cited in the Supplementary Materials.
