# Supplementary material for: UV-Crosslinked Poly(N-isopropylacrylamide) Interpenetrated into Chitosan Structure with Enhancement of Mechanical Properties Implemented as Anti-Fouling Materials
Source: Gels. 2023 Dec 25;10(1):20. doi: 10.3390/gels10010020 (PMC10815207; doi:10.3390/gels10010020)
Supplement: Supplementary file 1 [file gels-10-00020-s001.zip › gels-2759749-supplementary.pdf]

Supplementary Information

UV-crosslinked poly(*N*-isopropylacrylamide) interpenetrated into chitosan structure with enhancement of mechanical properties implementing as anti-fouling materials

Isala Dueramae[1, 2,*], Fumihiko Tanaka[3], Naoki Shinyashiki[1,4], Shin Yagihara[4] and Rio Kita[1,4,*]

[1]Micro/Nano Technology Center, Tokai University, Kanagawa 259-1292, Japan
[2]Metallurgy and Materials Research Institute, Chulalongkorn University, Bangkok, 10330, Thailand
[3]Department of Polymer Chemistry, Graduate School of Engineering, Kyoto University, Kyoto 615-8510, Japan
[4]Department of Physics, Tokai University, Kanagawa 259-1292, Japan
* Correspondence: author: isala.improve@gmail.com and rkita@keyaki.cc.u-tokai.ac.jp

1. Formation of single and interpenetrating polymer networks

A recent study aimed to enhance the properties of physical gel by altering the crosslinking agent in PNiPAM structure. Consequently, the composition and conditions utilized for the formation of chitosan hydrogel were arranged based on the previous reports [65,66], ensuring controlled development of the material. The CS hydrogel was prepared without purification using a modification of a previously reported method [65,66]. In brief, CS was dissolved in a 1.0% (w/v) acetic acid solution to a concentration of 1% by weight (wt%), which is close to the critical concentration for CS chain entanglement. The mixture was filtered through a 0.45-µm Millipore membrane. Then, the CS solution was mixed with 1, 3-propanediol at a 1:1 (v/v) ratio and poured into glass Petri dishes. The gel was obtained by evaporation of the solvent from the solution at 50°C for 3 days. Finally, the gel was thoroughly washed with deionized water to remove any remaining solvents. The formation structure of chitosan was proposed in Figure S1 (a).

The PNiPAM hydrogels were prepared using UV-irradiation. The initial reactants were dissolved in two different solvents: ethanol and deionized water (DI water). For example, 1 M of NiPAM monomer and 0.04 M (5.16wt%) of BIS crosslinking agent were dissolved in these solvents. The solution was stirred continuously for 1 hour at room temperature. After that, a fixed amount of I2965 photo-initiator was added and the mixture was stirred for an additional 30 minutes. The solution mixtures were bubbled with nitrogen for 5 minutes. Polymerization was carried out in a UV chamber using 365 nm of LUV-16 (Handy UV lamp), positioned on top of the chamber at a distance of 15 cm from the sample, for 3 hours. After polymerization, the hydrogels were washed with deionized water multiple times, and then dried at room temperature until a constant mass was achieved. The formation of PNiPAM was illustrated in Figure S1 (b). Two different solvents were used because the starting materials exhibited the highest solubility in DI water. However, when DI water was employed as the solvent, the studied properties showed no significant correlation with the independent parameter, namely the crosslinker content. As a result, ethanol was chosen as the primary solvent for this study.

2. Preparation of IPN hydrogels

A CS hydrogel with fixed dimensions was immersed in a solution containing the NiPAM monomer, crosslinker, and photo-initiator for 24 hours under various conditions, as shown in Table 1. Subsequently, it was irradiated with UV light, similar to pure PNiPAM hydrogels. After irradiation, the IPN sheets were washed with deionized water multiple times to remove any unreacted monomers and uncross-linked polymers. These sheets are then referred to as fresh hydrogel as shown in Figure S1 (c). The abbreviations

Citation: Dueramae, I.; Tanaka, F.; Shinyashiki, N.; Yagihara, S.; Kita, R. UV-crosslinked poly(*N*-isopropylacrylamide) interpenetrated into chitosan structure with enhancement of mechanical properties implementing as anti-fouling materials. *Gels* **2024**, *10*, 20. https://doi.org/10.3390/gels10010020

Academic Editors: John G. Hardy and Melike Firlak

Received: 22 November 2023
Revised: 16 December 2023
Accepted: 21 December 2023
Published: 25 December 2023

Copyright: © 2023 by the authors. Licensee MDPI, Basel, Switzerland. This article is an open access article distributed under the terms and conditions of the Creative Commons Attribution (CC BY) license (https://creativecommons.org/licenses/by/4.0/).

for the individual components and IPN were defined based on their composition and preparation solution, as indicated in Table1.

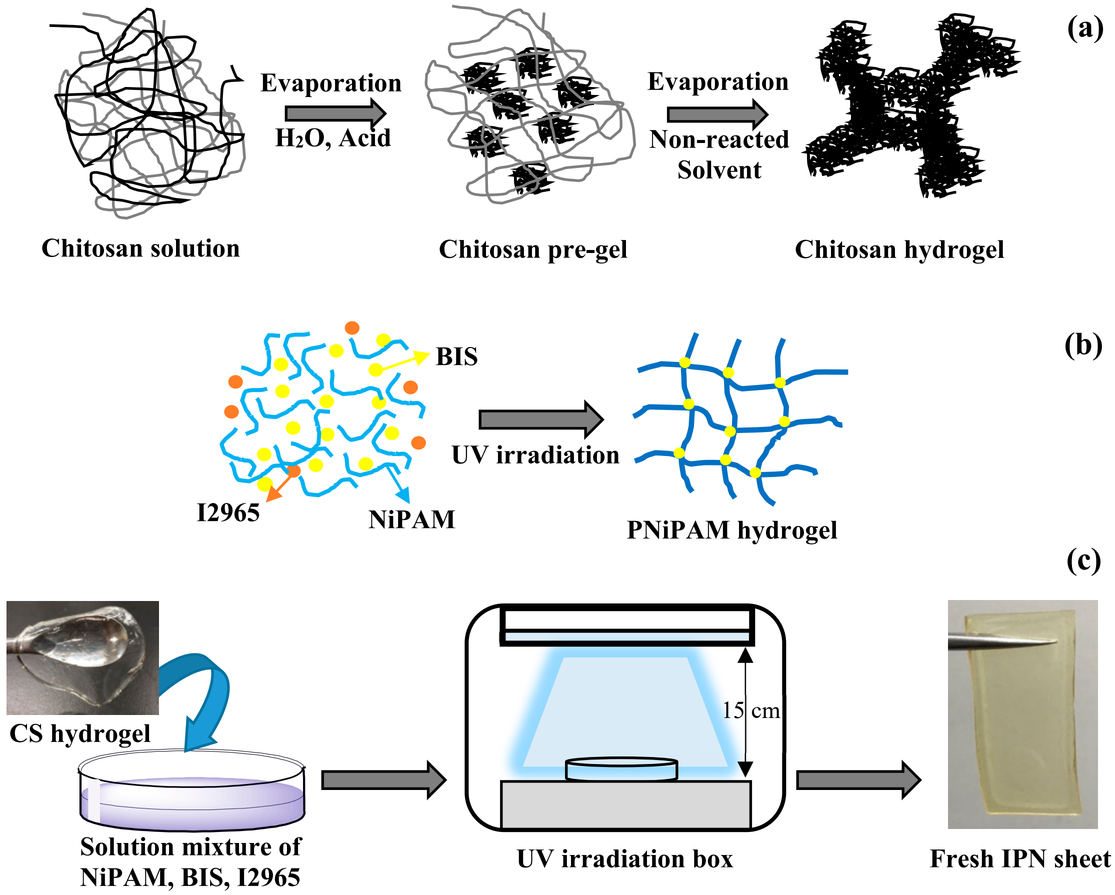

Figure S1. The fabrication process of (a) chitosan hydrogels, (b) PNiPAM hydrogels, and (c) the physical appearance of gels.

The zero-shear viscosity (η_0) is determined by analyzing the data of shear viscosities against shear rate for the ethanol solution of NiPAM monomer, crosslinker, and photo-initiator at different UV irradiation times, as shown in Figure S2(a), to identify the Newtonian plateau. Figure S2 (b) illustrates the frequency-dependent behavior of the shear modulus, G' and loss modulus, G'' for the ethanol solution of NiPAM monomer, crosslinker, and photo-initiator at different UV irradiation times. In the low ω region, G'' was greater than G', indicating a sol state of solutions for irradiation time 0 min (black). Conversely, in the high ω region, G' exceeded the loss modulus, G'' across almost the entire frequency range for the irradiation time of 240 min (cyan). However, G' exhibited a noticeable frequency dependence, particularly at the lower end of the frequency spectrum, suggesting the absence of a permanent crosslinked structure [67].

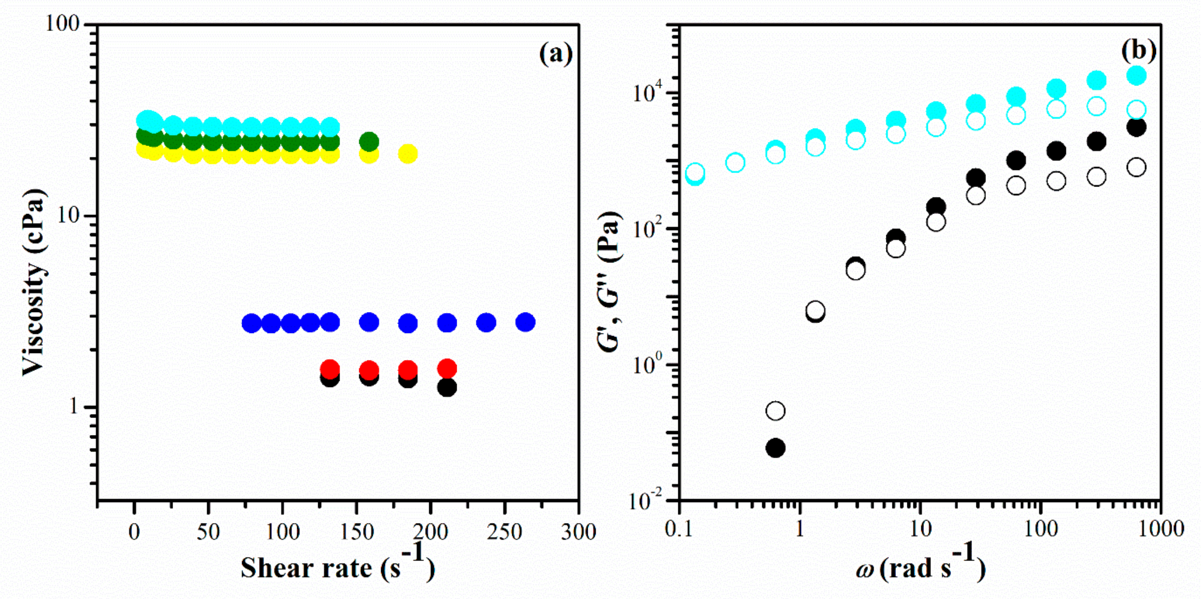

Figure S2. Rheological measurement of ethanol system of NiPAM monomer and photo-initiator containing BIS concentration of 1.45wt% at different UV irradiation times. (a) Shear-rate dependence of viscosity; UV irradiation times are 0 min (black), 30 min (red), 60 min (blue), 120 min (yellow), 180 min (olive), and 240 min (cyan). (b) Frequency dependence of *G*′ (filled circles) and *G*″ (hollow circles); UV irradiation times are 0 min (black) and 240 min (cyan).

Figure S3 illustrates the frequency dependence of shear modulus, *G*′, loss modulus, *G*″ of PNiPAME with a variation of BIS content at room temperature. *G*′ was significantly greater than *G*″, with both moduli showing slight variations as the frequency increased for all hydrogels except for PNiPAME hydrogel, containing BIS concentration of 1.48wt%. This observation suggests that the hydrogels possess a stable network with elastic characteristics, likely due to the strong molecular intra- and/or inter-actions.

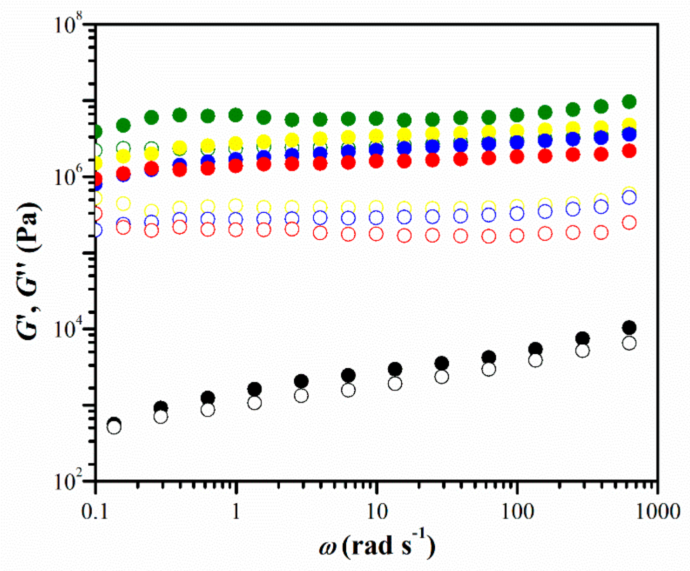

Figure S3. Frequency dependence of *G*′ (filled circles) and *G*″ (hollow circles) at room temperature for CS hydrogel (black) and PNiPAME hydrogels at various BIS concentrations as 1.48wt%(red), 2.65 wt% (blue), 5.16 wt% (olive), and 21.42 wt% (yellow).

The contribution of the covalent network of PNiPAM in the IPN structure was confirmed by the decline of tan δ with an increase in frequency, as seen in Figure S4, while that of chitosan tended to increase. The tan δ value should be zero for an ideal covalent gel. Therefore, these tan δ results indicate that the IPN gels include both chemically and physically cross-linked networks. The latter contributes to a viscous response to deformation. However, the viscous nature of the physical gels can provide the hydrogel with beneficial energy-damping capabilities across a wide frequency range.

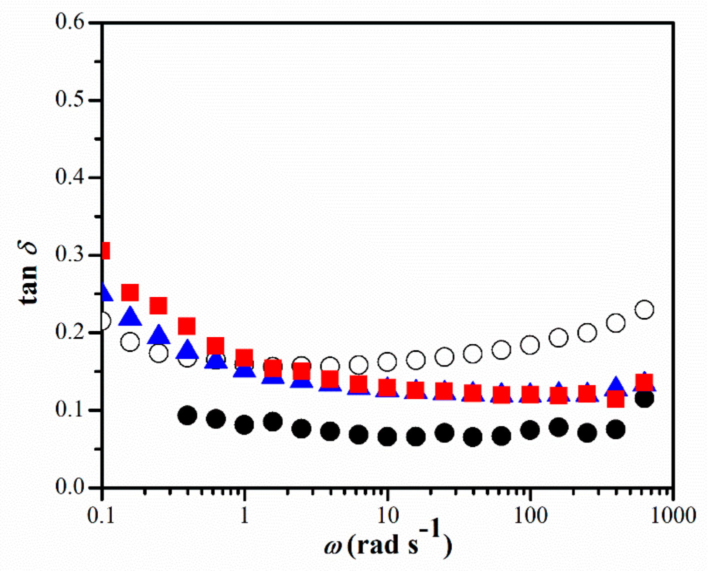

Figure S4. Frequency dependence of the tan δ for hydrogels of CS and IPNE series with various crosslinker content of CS hydrogel (0 wt% (○)), IPNE1 (1.45 wt% (●)), IPNE2 (2.65 wt.% (■)), and IPNE3 (5.16 wt.% (▲)) measured at room temperature.

As described in the experimental section, PNiPAM hydrogels were synthesized using NiPAM monomer, BIS crosslinking agent, and I2965 photo-initiator. By keeping all other factors constant, including the ratio of initial reactants and polymerization conditions, we produced transparent and opaque PNiPAM-based hydrogels using two different solvents: ethanol and deionized water (DI water), as depicted in Figure S5. Transparency is a desirable physical property in hydrogels for various biomedical applications, including antibacterial coatings and optical detectors for tissue engineering [68-70]. Conversely, opaque hydrogels are more commonly used as scaffolds for bone regeneration or bone tissue engineering [71]. Therefore, our PNiPAME system prepared using ethanol solution could be potentially utilized as anti-fouling materials in a biomedical field. In the previous studies, the transparent and opaque gels have also been reported by controlling all other parameters constant under different operating temperatures [72], pH [73], or initial cellulosic materials [74].

Furthermore, the quantity of solid gels formed depended on the crosslinker content. As depicted in Figure S6, the presence of solid or stable gels was observed by inverting the vial containing the prepared PNiPAME samples, as indicated by the area enclosed with the red dashed lines. The quantity of solid gels gradually increased with the rise in BIS concentration from 1.45wt% to 21.42wt%. It appears that the quantity of solid gels in PNiPAME with a BIS concentration of 21.42wt% is lower than that of 5.16wt%. However, it is evident that the former sample displayed translucent-like solid gels, suggesting a potentially higher crosslink density as discussed in section 2.2.

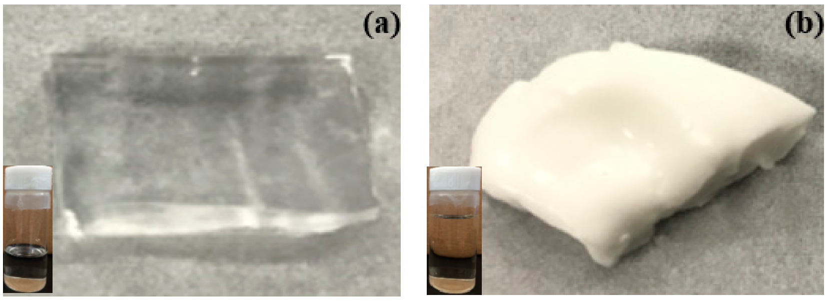

Figure S5. The physical appearance of PNiPAM hydrogels prepared in different solvents after UV irradiation for 3 hours; (a) ethanol and (b) deionized water. Insets show the transparency of NiPAM, BIS, and I2965 solution mixtures in both solvents before UV irradiation.

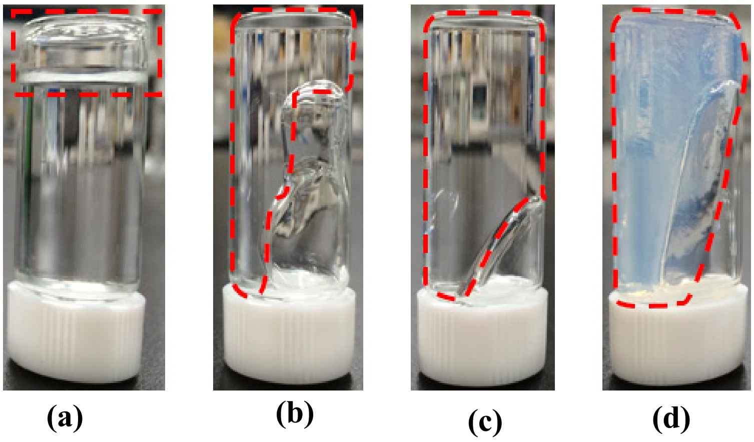

Figure S6. The physical appearance of PNiPAME hydrogels prepared in ethanol solvents with a crosslinker content of (a)1.45wt%, (b) 2.65 wt%, (c) 5.16 wt%, and (d) 21.42wt%.

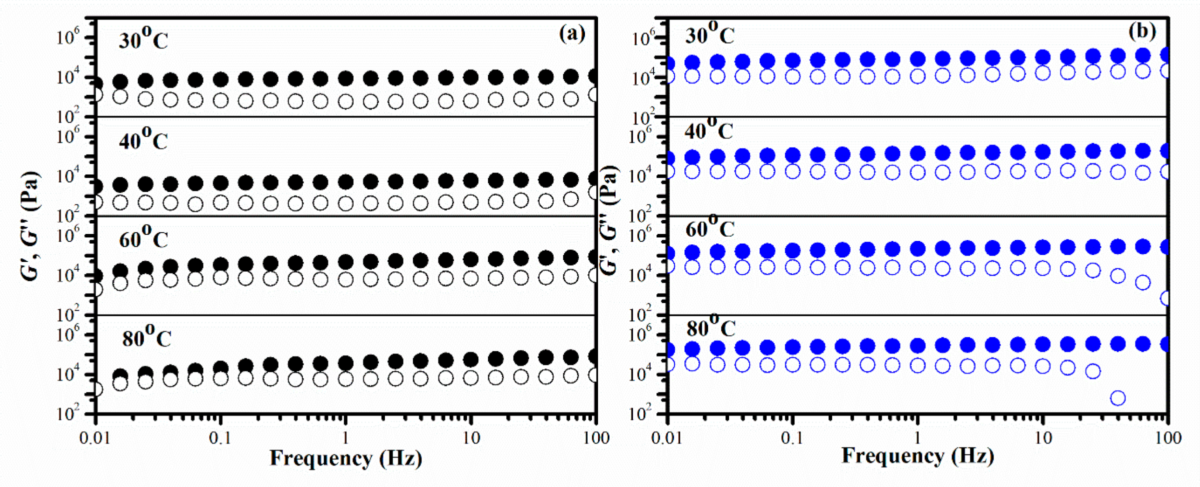

Figure S7. The frequency dependence on the *G*′ (filled circles) and *G*″ (hollow circles) of (a) CS (chitosan) and (b) IPNE3 hydrogels measured at different temperatures.

Figure S8 shows the *G*′ as a function of frequency for the system CS and IPNE3 hydrogels at different temperatures. The *G*′ of IPNE3 exhibited a higher magnitude compared to that of the CS hydrogel across the entire temperature range. The increase of *G*′ at 60 °C is attributed to the release of water from the CS network when the force and temperature were applied, resulting more rigid structure. However, the *G*′ decreased more rapidly as the frequency decreased, which can be attributed to the disruption of the physical network formed by hydrophobic associations. On the other hand, the IPNE3 exhibited

a more stable network in response to environmental changes, as indicated by the smaller slope of *G′* vs. *ω*. The increase in temperature causes *G′* to rise in IPN due to a subtle hydrophilic/hydrophobic phase transition within the network structure. In other words, this can be attributed to the enhanced of attractive interactions between the hydrogel networks, resulting from an increase in the hydrophobicity of the PNIPAM chains. At a temperature of 30 °C (T < VPTT), the hydrogel network swells with water due to the strong hydrogen bonds formed between the hydrophilic amide groups and water. This energy is higher than the unfavorable free energy resulting from the exposure of hydrophobic isopropyl groups to water, which ultimately leads to the formation of transparent gels. Above the VPTT, hydrophobic interactions are dominant, leading to the collapse of the polymer network due to water release [17]. This results in the formation of opaque gels, as shown in Figure S9 (b).

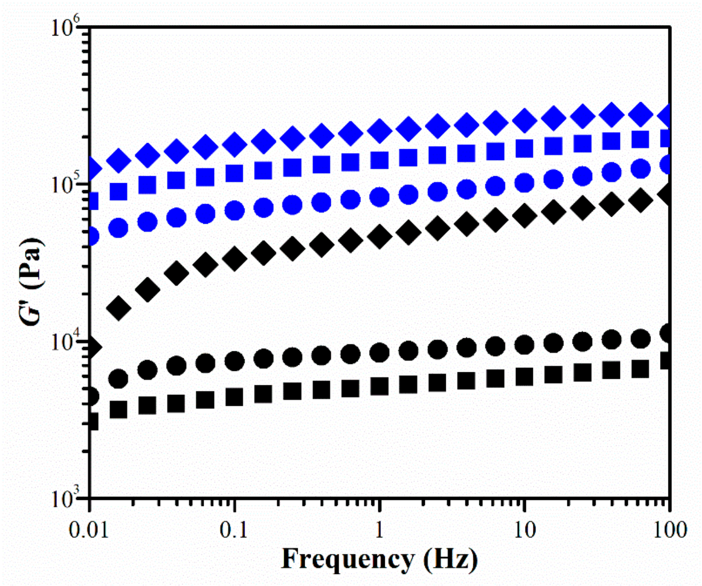

Figure S8. Frequency dependence on the shear modulus of the swollen state of CS (black) and IPN$_E$3 (blue) hydrogels at the temperatures 30°C (•), 40°C (■), and 60°C (♦).

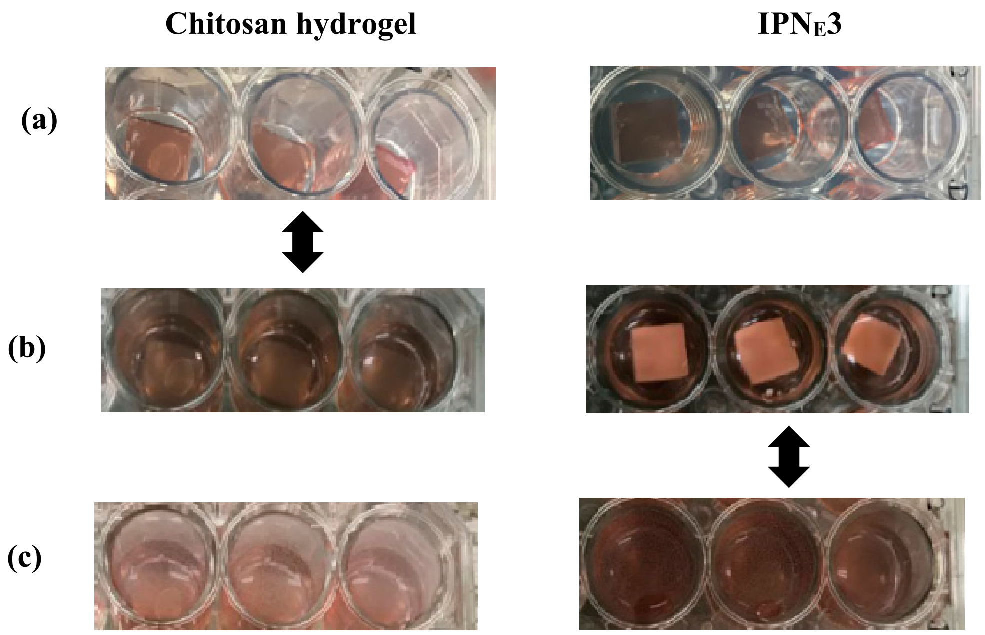

Figure S9. The physical appearance for hydrogels of CS (left side) and thermo-reversible behavior of IPN_E3 (right side). (a) At room temperature before cell seeding. (b) During the incubation process at 37 °C. (c) Room temperature after incubation process.
